# Differences in risk factors for self-harm with and without suicidal intent: Findings from the ALSPAC cohort

**DOI:** 10.1016/j.jad.2014.07.009

**Published:** 2014-10-15

**Authors:** Becky Mars, Jon Heron, Catherine Crane, Keith Hawton, Judi Kidger, Glyn Lewis, John Macleod, Kate Tilling, David Gunnell

**Affiliations:** a School of Social and Community Medicine, University of Bristol, United Kingdom; b Department of Psychiatry, University of Oxford, United Kingdom; c Mental Health Sciences Unit, University College London, United Kingdom

**Keywords:** ALSPAC, Adolescent, Self-harm, Suicide attempt, Longitudinal

## Abstract

**Background:**

There is a lack of consensus about whether self-harm with suicidal intent differs in aetiology and prognosis from non-suicidal self-harm, and whether they should be considered as different diagnostic categories.

**Method:**

Participants were 4799 members of the Avon Longitudinal Study of Parents and Children (ALSPAC), a UK population-based birth cohort who completed a postal questionnaire on self-harm with and without suicidal intent at age 16 years. Multinomial logistic regression analyses were used to examine differences in the risk factor profiles of individuals who self-harmed with and without suicidal intent.

**Results:**

Many risk factors were common to both behaviours, but associations were generally stronger in relation to suicidal self-harm. This was particularly true for mental health problems; compared to those with non-suicidal self-harm, those who had harmed with suicidal intent had an increased risk of depression (OR 3.50[95% CI 1.64, 7.43]) and anxiety disorder (OR 3.50[95% CI 1.72, 7.13]). Higher IQ and maternal education were risk factors for non-suicidal self-harm but not suicidal self-harm. Risk factors that appeared specific to suicidal self-harm included lower IQ and socioeconomic position, physical cruelty to children in the household and parental self-harm.

**Limitations:**

i) There was some loss to follow-up, ii) difficulty in measuring suicidal intent, iii) we cannot rule out the possibility of reverse causation for some exposure variables, iv) we were unable to identify the subgroup that had only ever harmed with suicidal intent.

**Conclusion:**

Self-harm with and without suicidal intent are overlapping behaviours but with some distinct characteristics, indicating the importance of fully exploring vulnerability factors, motivations, and intentions in adolescents who self harm.

## 1 Introduction

Adolescent self-harm is a major public health concern, with community studies reporting a lifetime risk of 13–18% (Kidger et al., 2012, Evans et al., 2005, Hawton et al., 2002). It is not only a signal of an individual׳s distress but is also the strongest risk factor for later suicide (Hawton et al., 2003).

Despite increased awareness of the importance of self-harm (NICE, 2011); there remains a lack of consensus over how it should be conceptualised. Some researchers argue that a clear distinction can be made between acts of self-harm that occur with intent to die (suicide attempts [SA]) and those that occur with no intent to die (e.g. Non-Suicidal Self-Injury [NSSI]) (Muehlenkamp and Kerr, 2010, Nock, 2010). Moreover, NSSI has now been included under ‘conditions for further study’ in the fifth edition of the Diagnostic and Statistical Manual (DSM-V).

Several important differences have been found between SA and NSSI including differences in prevalence, frequency, lethality of methods, and attitudes towards life and death (Muehlenkamp and Kerr, 2010). However, the considerable overlap between self-harm with and without suicidal intent, including the fact that many individuals engage in both behaviours (Hamza et al., 2012, Jacobson et al., 2008, Wilkinson et al., 2011, Klonsky et al., 2013) has led some researchers to argue that they are best conceptualised along a continuum (Kapur et al., 2013, Stanley et al., 1992).

Many previous investigations of adolescent self-harm have focused on clinically presenting samples which account for <20% of all episodes (Kidger et al., 2012, Hawton et al., 2002). Moreover, the majority of studies have either not distinguished individuals according to suicidal intent, or have focused on one behaviour only.

In a clinical sample of adolescents undergoing treatment for depression, Wilkinson et al. (2011) found a different pattern of risk factors for future NSSI and SA over 28 week follow-up. Both NSSI and SA were predicted by previous NSSI, however, future SA was additionally predicted by poor family functioning and future NSSI by hopelessness, anxiety disorder, younger age and female gender. In a Hong Kong sample of students reporting self-harm (Wong et al., 2007), those who had attempted suicide in the previous 12 months were found to have higher depression, anxiety and substance use scores, as well as greater life stress and poorer family relationships than those with non-suicidal self-harm. There is also some evidence to suggest that risk factors may differ according to gender (Hargus et al., 2009).

There have been few longitudinal population studies investigating self-harm with and without suicidal intent. In a five-year prospective study of Norwegian high-school students (Wichstrøm, 2009), a number of common risk and protective factors were identified, but several others were associated with only one or other behaviour. Self-harm without suicidal intent was associated with previous non-suicidal self-harm, young age of first engagement in sexual activity and low satisfaction with social support whereas self-harm with suicidal intent was associated with suicidal ideation, conduct problems and low levels of parental care. In another Norwegian sample (Larsson and Sund, 2008), adolescents who attempted suicide over the 1 year follow-up reported higher depression scores, higher internalising, externalising and total problem scores and more often knew a friend who had attempted/died by suicide than those who reported self-harm without suicidal intent.

Taken together, these studies suggest that there may be important differences between self-harm with and without suicidal intent; however findings across studies have failed to produce clear conclusions. The present study extends previous research by examining risk factors for adolescent self-harm in a large UK population-based birth cohort. Individuals reporting self-harm with and without suicidal intent were compared on a wide range of recognised risk factors, in order to investigate whether risk factors for self-harm with and without suicidal intent differ.

## 2 Methods

### 2.1 Sample

The Avon Longitudinal Study of Parents and Children (ALSPAC) is an ongoing population-based birth cohort study examining influences on health and development across the lifecourse. The ALSPAC core enroled sample consists of 14,541 pregnant women resident in the former county of Avon in South West England (United Kingdom), with expected delivery dates between 1st April 1991 and 31st December 1992 (Boyd et al., 2013). Of 14,062 live births, 13,798 were singletons/first-born of twins and were alive at one year of age. Participants have been followed-up regularly since recruitment through questionnaires and research clinics. Detailed information about ALSPAC is available on the study website (http://www.bristol.ac.uk/alspac), which includes a fully searchable data-dictionary of available data (http://www.bris.ac.uk/alspac/researchers/data-access/data-dictionary). Ethical approval for the study was obtained from the ALSPAC Law and Ethics committee and local research ethics committees. Written informed consent was obtained after the procedure(s) had been fully explained.

**Fig. 1:**
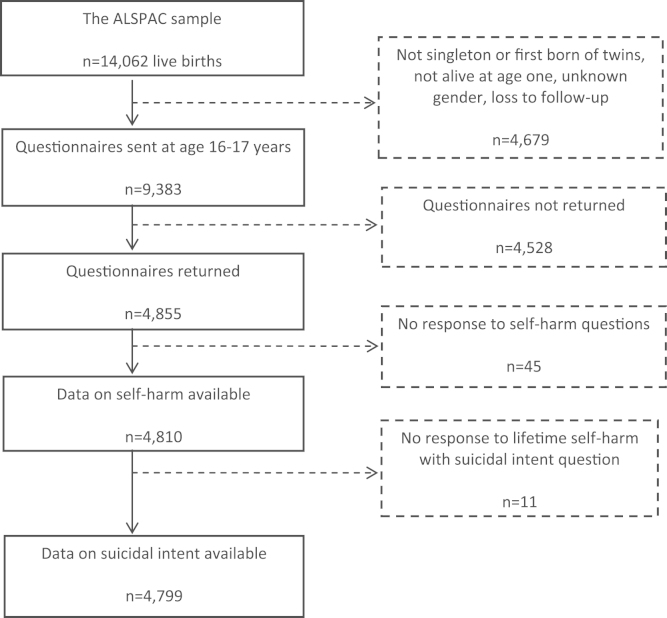
Flow-chart of attrition and self-harm outcomes In the Avon Longitudinal Study of Parents and Children (ALSPAC) birth cohort.

The present investigation is based on 4799 children who completed a detailed self-harm questionnaire at age 16 years (Fig. 1) (Kidger et al., 2012). Compared with non-responders (*n*=4528), those who returned the questionnaire (*n*=4855) were more likely to be females, have a mother in a non-manual social class and have relatively high educational qualifications (Kidger et al., 2012). Information about suicidal intent was missing for 11 respondents.

### 2.2 Measures

#### 2.2.1 Outcome measure: self-harm

The self-harm questions included in the ALSPAC questionnaire were based on those used in the Child and Adolescent Self-harm in Europe (CASE) study (Madge et al., 2008). Participants who responded positively to the item “have you ever hurt yourself on purpose in any way (e.g. by taking an overdose of pills or by cutting yourself)?” were classified as having a history of self-harm. An additional two questions were used to identify participants who had self-harmed with suicidal intent. Participants were classified as having self-harmed with suicidal intent if i) they responded to the question “Do any of the following reasons help to explain why you hurt yourself on that (i.e. the most recent) occasion?” by selecting the option “I wanted to die” or ii) they responded positively to the question “On any of the occasions when you have hurt yourself on purpose, have you ever seriously wanted to kill yourself?”.

These questions allowed us to identify individuals who had harmed with suicidal intent at some point during their lifetime, and those who had only ever engaged in non-suicidal self-harm. However, individuals may self-harm on multiple occasions, and population studies suggest that between 79% and 93% of adolescents who have attempted suicide have also harmed without suicidal intent (Muehlenkamp and Gutierrez, 2007, Brausch and Gutierrez, 2010, Zetterqvist et al., 2013). Throughout the paper, we refer to those with a lifetime history of suicidal self-harm as having ‘harmed with suicidal intent’, but recognise that many individuals in this group have also engaged in episodes of non-suicidal self-harm. We were unable to identify the proportion of adolescents that have engaged in both behaviours, but the *minimum* degree of overlap in this sample is 30% (i.e. 30% of participants who had harmed with suicidal intent during their lifetime reported their most recent episode was non-suicidal).

Questions have been raised about the reliability of measuring suicidal intention as reports may be biased by current mood state, individuals may have been ambivalent, or may not truly intend to end their life. In accordance with previous research (Nock, 2010) the approach used in this study was to classify self-harm behaviours according to individual׳s self-reported suicidal intent.

#### 2.2.2 Exposure variables

Exposure variables were selected on the basis that they are widely recognised risk factors for self-harm and had been previously recorded on study members.

*IQ and socioeconomic position:* Child IQ, assessed using the Wechsler Intelligence Scale for Children (WISC-III) (Wechsler, 1991) at age eight years and measures of parent׳s socioeconomic position, obtained from maternal questionnaires including i) average weekly household disposable income recorded at age 3 and 4 years, divided into quintiles and re-scaled to account for family size, composition and estimated housing benefits (Gregg et al., 2008); ii) social class (professional/managerial or other) identified during pregnancy, (the highest of maternal or paternal social class was used); and iii) highest maternal educational attainment (less than O-level, O-level, A-Level or university degree) measured during pregnancy (O-levels and A-levels are school qualifications taken around age 16 and 18 years, respectively).

*Early adverse experiences:* Childhood sexual abuse (source: maternal questionnaires, repeated seven times from birth-eight years); physical cruelty to children in the household by mother/partner (source: maternal questionnaires, repeated eight times from birth-11 years); and bullying/victimisation (overt or relational bullying at least once a week over the previous 6 months), assessed using a modified version of the bullying and friendship interview schedule (Woods and Wolke, 2003) at age 12 years.

*Mental health, personality and behaviour:* Impulsivity, assessed using the stop-signal task (mean number of correct trials) (Logan et al., 1984), at age ten years; sensation-seeking, assessed using the novelty and intensity subscales of the Arnett Inventory of Sensation-Seeking scale (Arnett, 1994) at age 16 years; body dissatisfaction (unhappy or happy over the past year), identified at age 13 years; child depression symptoms, assessed using the Short Mood and Feelings Questionnaire (Angold et al., 1995) at age 14 years (scores of 11+ were used to indicate significant depression symptoms) (Patton et al., 2008); depressive and anxiety disorder, assessed using the semi-structured DAWBA interview (Goodman et al., 2000) at age 15 years; substance use, identified at age 15 years including heavy alcohol use (consuming >4 drinks on a typical occasion in the last six months), cannabis use (at least occasional use) and regular smoking (at least weekly).

*Exposure to self-harm:* Self-harm in friends, mother and father, reported by the child at age 16 years and parental suicide attempt (source: maternal questionnaires, repeated eight times from birth-11 years).

### 2.3 Statistical analysis

Multinomial regression was used to examine associations with exposure variables in relation to a three-category self-harm outcome: no self-harm; self-harm without suicidal intent; and self-harm with suicidal intent. Analyses were adjusted only for participant gender as our aim was to identify potential differences in risk factors for self-harm with and without suicidal intent, rather than to build the most parsimonious prediction model. Exposures with more than two categories were treated as linear unless there was evidence for a departure from a linear relationship. Due to the well recognised gender differences in the incidence of self-harm (Evans et al., 2005), secondary analyses formally tested exposure-gender interactions using likelihood ratio tests. All analyses were conducted using Stata version 12.

*Missing data imputation:* Primary analyses were conducted on an imputed dataset based on those with complete outcome data (*n*=4799). Multivariable Imputation by Chained Equations (Royston and White, 2011) in Stata was used to create multiple copies of datasets in which missing covariate data are replaced by imputed values, sampled from their predictive distribution. This method assumes that data are Missing at Random (MAR), whereby any systematic differences between the missing and the observed values can be explained by differences in observed data (Sterne et al., 2009).

Two hundred imputed datasets were generated. All variables used in the analysis were included in the imputation models along with a number of additional auxiliary variables. These included indicators of socioeconomic adversity, maternal psychopathology and demographics as well as other measures of the exposure variables collected earlier/later in the study. Imputations were generated separately for males and females to allow for possible gender interactions. Monte Carlo errors are available on request.

## 3 Results

Of 4799 participants with data on self-harm up to age 16 years, 569 (11.9% [95% CI 10.9%–12.8%]) reported self-harm without suicidal intent, but no episodes of suicidal self-harm, and 325 (6.8% [95% CI 6.1%–7.5%]) reported self-harming with suicidal intent on at least one occasion. Participants who had self-harmed with suicidal intent at some point during their lifetime were more likely than those who self-harmed without suicidal intent to have taken an overdose on the most recent occasion (28% vs. 5%, risk difference 0.23 [95% CI 0.17, 0.28]) and to have sought medical help (55% vs. 19%, risk difference 0.36 [95% CI 0.30, 0.43]). There was little evidence of group differences in using cutting as a method of self-harm on the most recent occasion (84% vs. 83%, risk difference 0.01 [95% CI −0.04, 0.06]).

Females were more likely to report self-harm than males; 81.2% and 79.4% of those who had self-harmed with and without suicidal intent were females (Table 1). The self-harming groups generally had higher levels of risk factors than those who had not self-harmed. Risk factors were also generally higher/more prevalent amongst those who had self-harmed with suicidal intent than without, although the self-harm groups were similar with regards to gender, sensation-seeking and self-harm in friends. Those with non-suicidal self-harm had higher mean IQ scores and greater prevalence of cannabis and heavy alcohol use than those with suicidal self-harm (Table 1).

**Table 1:** Descriptive table for key exposure variables.

**Exposure (age of assessment)**	**No self-harm (*****n*****=3905)**		**Self-harm without suicidal intent (*****n*****=569)**		**Self-harm with suicidal intent (*****n*****=325)**		***P***-**value**⁎
Female gender, *n* (%)	2113	(54.1%)	452	(79.4%)	264	(81.2%)	<0.001

Socioeconomic position							
*Equivalised income (*33 and 47 *months)*, *n* (%)^a^							0.003
5th quintile (lowest)	482	(13.6%)	70	(13.5%)	50	(16.9%)	
4th quintile	597	(16.8%)	90	(17.3%)	74	(25.0%)	
3rd quintile	694	(19.5%)	113	(21.7%)	58	(19.6%)	
2nd quintile	832	(23.4%)	117	(22.5%)	61	(20.6%)	
1st quintile (highest)	949	(26.7%)	130	(25.0%)	53	(17.9%)	
*Parent social class (pregnancy)*, *n* (%)^b^							0.004
Other	1262	(34.6%)	184	(34.5%)	130	(44.1%)	
Professional/managerial	2390	(65.4%)	349	(65.5%)	165	(55.9%)	
*Mother׳s education (pregnancy)*, *n* (%)							0.001
<O-level	712	(18.7%)	87	(15.7%)	70	(22.3%)	
O-level	1236	(32.5%)	204	(36.8%)	112	(35.7%)	
A level	1085	(28.5%)	131	(23.6%)	90	(28.7%)	
Degree	771	(20.3%)	133	(24.0%)	42	(13.4%)	

Total IQ (age 8), mean (SD)	107.31	(16.3)	109.83	(14.8)	105.05	(16.6)	<0.001
Sexual abuse (birth - age 8), *n* (%)	16	(0.5%)	5	(1.0%)	4	(1.4%)	0.056
Parental cruelty to children (birth- age 11), *n* (%)	101	(3.8%)	19	(4.9%)	24	(11.4%)	<0.001
Being bullied (age 12), *n* (%)	716	(23.3%)	138	(30.3%)	97	(40.3%)	<0.001
Impulsivity (age 10), stop-signal task, mean number of trials correct at 250 ms delay (SD)	13.70	(2.6)	13.74	(2.5)	13.49	(2.7)	0.407

Sensation-seeking (age 16), mean (SD)							
*Arnett intensity subscale*	25.75	(4.5)	26.19	(4.8)	26.24	(4.5)	0.028
*Arnett novelty subscale*	25.71	(4.3)	26.63	(4.4)	25.73	(4.6)	<0.001

Body dissatisfaction (age 13), *n* (%)	926	(28.6%)	228	(47.6%)	153	(56.9%)	<0.001

Mental health							
*Depressive symptoms* (*age* 14)*, SMFQ score* 11*+, n* (%)	232	(7.9%)	92	(21.2%)	76	(33.3%)	<0.001
*Depressive disorder* (*age* 15)*, DAWBA, n* (%)	23	(0.8%)	11	(2.7%)	18	(8.3%)	<0.001
*Anxiety disorder* (*age* 15)*, DAWBA, n* (%)	27	(1.0%)	12	(2.9%)	18	(8.3%)	<0.001

Substance use (age 15)							
*Alcohol, heavy drinking, n* (%)	450	(16.7%)	119	(29.1%)	54	(25.7%)	<0.001
*Cannabis, at least occasional use, n* (%)	174	(6.2%)	73	(17.6%)	31	(14.4%)	<0.001
*Smoking, at least weekly, n* (%)	157	(5.6%)	50	(12.1%)	52	(24.1%)	<0.001

Self-harm in friends and family							
*Parent suicide attempt* (*birth-age* 11)*, n* (%)	43	(1.2%)	5	(1.0%)	14	(4.9%)	<0.001
*Mother self-harm* (*age* 16)*, n* (%))^c^	26	(0.7%)	15	(2.7%)	28	(8.7%)	<0.001
*Father self-harm* (*age* 16)*, n* (%) c	20	(0.5%)	5	(0.9%)	8	(2.5%)	<0.001
*Self-harm in friends* (*age* 16)*, n* (%) c	1202	(31.0%)	428	(75.8%)	261	(80.6%)	<0.001

SMFQ: short mood and feelings questionnaire.

Number of respondents with missing data was 0 for gender; 429 for income; 320 for social class; 126 for maternal education; 984 for total IQ; 452 for sexual abuse; 1574 for physically cruel to children; 1024 for being bullied; 1142 for impulsivity; 129 for the intensity subscale of the Arnett׳s Sensation-seeking scale; 148 for the novelty subscale of the Arnett׳s Sensation-seeking scale; 807 for body dissatisfaction; 1205 for SMFQ score; 1339 for DAWBA depression; 1338 for DAWBA anxiety; 1477 for heavy alcohol; 1383 for cannabis; 1362 for smoking; 28 for self-harm in friends (child rated); 540 for parent suicide attempt (parent rated); 28 for mother self-harm (child rated) and 28 for father self-harm (child rated).

⁎ Chi-Square test of the association between self-harm and categorical exposures and ANOVA for differences in means for continuous exposures.

a Quintiles represent lowest to highest household income. Quintiles were derived from income measures at ages 33 and 47 months on a larger subset of the cohort, and so in the present sample numbers are not evenly distributed.

b Highest social class of mother and father.

c Child-rated.

### 3.1 Risk factors for self-harm with and without suicidal intent

**Fig. 2:**
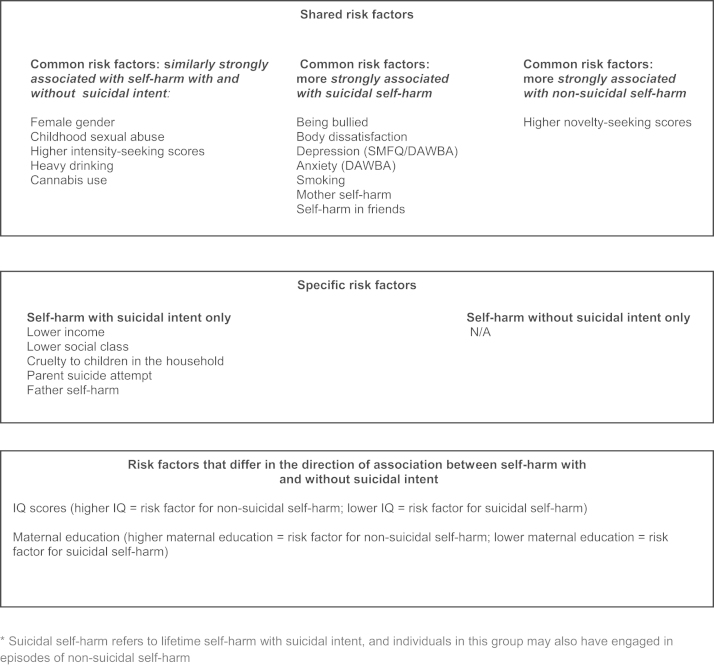
Shared, specific and differing risk factors for self-harm with and without suicidal intent.

Associations between exposure variables and adolescent self-harm with and without suicidal intent are presented in the first two columns of ORs in Table 2 and summarised in Fig. 2; the reference group for these ORs are adolescents who have never self-harmed. Findings were very similar in the complete case analysis (see Supplementary Table 1). Differences between those who self-harmed with and without suicidal intent are shown in the third column of ORs. In this column, ORs>1 indicate that a particular exposure is more strongly associated with suicidal than non-suicidal self-harm; ORs<1.0 indicate the reverse. The omnibus *P*-values (column 1) give the statistical evidence against the null hypothesis of no association between each risk factor and any category of self-harm (with or without suicidal intent).

**Table 2:** Associations between exposures and self-harm with and without suicidal intent (*n*=4799).

**Exposure**	**Omnibus test for exposure**	**Self-harm without suicidal intent versus no self-harm**	**Self-harm with suicidal intent versus no self-harm**	**Self-harm with suicidal intent versus self-harm without suicidal intent**
	***P***	**OR [95% CI]**	**OR [95% CI]**	**OR [95% CI]**
Female gender	<0.001	3.28 [2.65, 4.05]	3.68 [2.77, 4.90]	1.12 [0.80, 1.58]
				
Socioeconomic position				
*Equivalised income* (*per quintile, 0* [*high*] −4 [*low*])	<0.001	1.01 [0.95, 1.08]	1.21 [1.11, 1.31]	1.19 [1.07, 1.32]
*Parent social class* (*other vs. professional/managerial*)	0.007	0.95 [0.78, 1.15]	1.44 [1.13, 1.83]	1.52 [1.14, 2.03]
*Mothers education* (*degree=reference*)	0.001			
*A level*		0.69 [0.53, 0.90]	1.48 [1.01, 2.17]	2.14 [1.38, 3.32]
*O-level*		0.92 [0.73, 1.17]	1.59 [1.10, 2.30]	1.72 [1.14, 2.62]
<*O-level*		0.65 [0.48, 0.86]	1.66 [1.12, 2.47]	2.57 [1.61, 4.09]
				
Total IQ (10 point increments)	<0.001	1.14 [1.07, 1.21]	0.92 [0.85, 0.99]	0.81 [0.74, 0.89]
Childhood sexual abuse	0.031	2.32 [0.89, 6.01]	3.54 [1.24, 10.1]	1.53 [0.45, 5.17]
Cruelty to children in household	<0.001	1.38 [0.85, 2.24]	3.26 [2.09, 5.09]	2.36 [1.32, 4.24]
Being bullied	<0.001	1.49 [1.19, 1.85]	2.41 [1.85, 3.14]	1.62 [1.18, 2.22]
Impulsivity (stop-signal task)	0.326	1.00 [0.96, 1.05]	0.97 [0.92, 1.01]	0.96 [0.91, 1.02]
				
Sensation-seeking (5 point increments)				
*Arnett intensity subscale*	<0.001	1.48 [1.32, 1.65]	1.54 [1.33, 1.77]	1.04 [0.88, 1.23]
*Arnett novelty subscale*	<0.001	1.43 [1.28, 1.59]	1.13 [0.99, 1.29]	0.79 [0.67, 0.93]
				
Body dissatisfaction	<0.001	1.92 [1.57, 2.35]	2.84 [2.20, 3.66]	1.48 [1.09, 1.99]

Mental health				
*Depressive symptoms* (*SMFQ* 11*+*)	<0.001	2.63 [2.03, 3.40]	4.97 [3.70, 6.69]	1.89 [1.34, 2.66]
*DAWBA depression*	<0.001	2.14 [1.06, 4.30]	7.47 [4.10, 13.6]	3.50 [1.64, 7.43]
*DAWBA anxiety*	<0.001	2.06 [1.08, 3.92]	7.20 [4.07, 12.7]	3.50 [1.72, 7.13]

Substance use				
*Alcohol* (*heavy drinking*)	<0.001	1.92 [1.52, 2.43]	1.71 [1.25, 2.34]	0.89 [0.62, 1.28]
*Cannabis* (*occasional*)	<0.001	3.21 [2.38, 4.33]	2.38 [1.62, 3.51]	0.74 [0.48, 1.14]
*Smoking* (*weekly*)	<0.001	1.59 [1.17, 2.16]	3.51 [2.53, 4.88]	2.21 [1.49, 3.29]

Self-harm in friends and family				
*Parent suicide attempt*	<0.001	0.90 [0.36, 2.24]	4.24 [2.31, 7.81]	4.74 [1.73, 13.0]
*Mother self-harm* (*child-rated*)	<0.001	3.41 [1.78, 6.55]	11.9 [6.82, 20.9]	3.50 [1.84, 6.66]
*Father self-harm* (*child-rated*)	0.004	1.50 [0.55, 4.07]	4.26 [1.83, 9.93]	2.84 [0.92, 8.75]
*Friend self-harm* (*child-rated*)	<0.001	5.86 [4.75, 7.22]	7.70 [5.76, 10.3]	1.31 [0.93, 1.85]

All analyses adjusted for participant gender.

SMFQ: short mood and feelings questionnaire.

The omnibus *P*-values (column 1) give the statistical evidence against the null hypothesis of no association between each risk factor and any category of self-harm (with or without suicidal intent). The first two column of OR׳s give the associations between exposure variables and adolescent self-harm with and without suicidal intent; the reference group for these ORs are adolescents who have never self-harmed. Differences between those who self-harmed with and without suicidal intent are shown in the third column of ORs. In this column, ORs>1 indicate a particular exposure is more strongly associated with suicidal than non-suicidal self-harm; ORs<1.0 indicate the reverse.

### 3.2 Exposures similarly strongly associated with both self-harm groups

Females were over three times more likely to report having self-harmed than males for both categories of self-harm; this pattern of risk did not differ between self-harm with and without suicidal intent (OR 1.12 [95% CI 0.80, 1.58]). Other exposures that were similarly strongly associated with an increased risk of both self-harm with and without suicidal intent were childhood sexual abuse, higher intensity-seeking scores, heavy drinking and cannabis use. Impulsivity, as indexed by the stop-signal task, was the only exposure that showed little evidence of an association with either self-harm group.

### 3.3 Exposures that differ according to self-harm group

Socioeconomic position (lower income and social class), cruelty to children in the household, parent suicide attempt and paternal self-harm appear to be specific risk factors for self-harm with suicidal intent, as there was little evidence of an association with non-suicidal self-harm. For IQ and maternal education a different pattern of association was found across the two self-harm groups; higher IQ and higher levels of maternal education were associated with an increased risk of self-harm without suicidal intent but a reduced risk of self-harm with suicidal intent.

Most other risk factors were associated with both self-harm behaviours, but associations were generally stronger in relation to suicidal self-harm. This was particularly pronounced for mental health problems (DAWBA depression OR 3.50 [95% CI 1.64, 7.43]; DAWBA anxiety disorder OR 3.50 [95% CI 1.72, 7.13]). Associations with novelty seeking scores were stronger for self-harm without suicidal intent than with suicidal intent (OR 0.79 [95% CI 0.67, 0.93]).

### 3.4 Gender interactions

In secondary analyses, likelihood ratio tests were conducted to examine potential differences according to child gender (Supplementary Table 2). Strong evidence (*P*<0.01) was found for only two exposure variables: smoking and self-harm in friends. Smoking was associated with a substantially greater risk of self-harm with suicidal intent for females than for males. For girls, smoking was associated with a six-fold increase in odds of suicidal self-harm (OR 6.09 [95% CI 4.13, 9.00]), however there was little evidence of an association in boys (OR 0.93 [95% CI 0.22, 3.95]).

Self-harm in friends was associated with a substantially higher risk of both self-harm behaviours for males than for females. For boys, self-harm in friends was associated with a nine-fold increase in odds of non-suicidal self-harm (OR 9.07, 95% CI 6.07, 13.5), whereas for girls the odds were increased five-fold (OR 4.93, 95% CI 3.88, 6.28). For suicidal self-harm, friend self-harm was associated with 13-fold increase in odds for boys (OR 13.2, 95% CI 7.39, 23.7), over double the odds found for girls (OR 6.27, 95% CI 4.52, 8.69).

## 4 Discussion

### 4.1 Main findings

The present study is the first to examine risk factors for self-harm with and without lifetime suicidal intent in a large, population-based birth cohort. Both common and specific risk factors were identified, suggesting that these are overlapping behaviours, with some distinct characteristics. The presence of common risk factors is indicative both of a shared vulnerability and could also reflect the high rate of crossover between the groups; a minimum of 30% of those in the suicidal self-harm group also engaged in acts of non-suicidal self-harm. However, while these behaviours clearly overlap, for several risk factors there was evidence of differences in either the magnitude or the direction of the association across the two self-harm groups, suggesting that there may also be some differences in their aetiology. As found previously in this sample (Chang et al., 2014) higher IQ, and also maternal education appear to be specifically associated with an increased risk of non-suicidal self-harm. Indeed, these variables were associated with a decreased risk of self-harm with suicidal intent. In contrast lower socioeconomic position, cruelty to children in the household, parent suicide attempt and paternal self-harm appear to be specifically associated with suicidal self-harm.

One possible explanation for the differences in the associations of IQ with self-harm with and without suicidal intent is that children from families where parents have higher levels of education (and so themselves are likely to have higher IQ) may feel under pressure to perform well in school, and use non-suicidal self-harm as a coping mechanism. Alternatively, it is possible that IQ might influence responses to the self-harm questions. The stronger association found between novelty seeking and non-suicidal self-harm could suggest that those with higher novelty seeking scores may be more likely to experiment with alternative ways of coping with stress.

In line with Hargus et al. (2009), we identified some gender differences in risk factors for self-harm, most notably for smoking where associations were stronger for girls and self-harm in friends where associations were stronger for boys. Similar gender differences have been found for peer effects on college drinking behaviour (Duncan et al., 2005) and for smoking on depression (Lien et al., 2009). However, gender interactions in the present sample need to be interpreted with caution, given the large number of tests conducted and the small number for some analyses.

### 4.2 Strengths and limitations

ALSPAC is a large population-based sample, which is important given that the majority of self-harm does not present to specialist services (Kidger et al., 2012, Hawton et al., 2002). A wide range of recognised risk factors were examined, using detailed prospectively recorded measures.

The findings must also be interpreted in light of several limitations. First, the loss to follow-up and questionnaire non-response may have led to selection bias, however results from our imputation models suggest that missing data had little effect on risk factor associations (Supplementary Table 1). Second, with the exception of sensation seeking and self-harm in friends and family, data on all exposures were collected prior to the self-harm questionnaire. However, as the age of self-harm onset is not known, it is possible that some exposures may have been measured subsequent to the first self-harm episode and we therefore cannot rule out the possibility of reverse causation. This is particularity salient for exposures assessed closest in time to the self-harm questionnaire, although we have no reason to believe that associations with self-harm with and without suicidal intent would be differentially effected. Reverse causation is unlikely to be a factor for those variables assessed before the age of 12 years, (SEP, IQ, sexual abuse, parental cruelty, impulsivity and parent suicide attempt), as self-harm before this age is rare.

A third limitation concerns the difficulty in measuring suicidal intent. In line with previous research (Nock, 2010), individuals were classified according to self-reported lifetime suicidal intent, however reports may be affected by current mood state. We found 22% (*n*=49) of adolescents who reported wanting to die on the most recent occasion responded negatively to the later question “have you ever seriously wanted to kill yourself”. For this group, self-harm may have been an expression of distress, rather than a reflection of suicidal intention, however additional analyses excluding these individuals revealed a similar pattern of results (available on request). Participants who had self-harmed with suicidal intent were more likely to use overdose as a method and to have sought help, providing some support for the distinction between the groups.

Fourth, individuals were classified as having harmed with suicidal intent if they had ever reported an act of suicidal self-harm during their lifetime. Many in this group have also engaged in episodes of non-suicidal self-harm, however, we were not able to distinguish between those who had only ever harmed with intent and those who had engaged in both behaviours. Previous population studies have found that between 79% and 93% of adolescents who have attempted suicide have also harmed without suicidal intent (Muehlenkamp and Gutierrez, 2007, Brausch and Gutierrez, 2010, Zetterqvist et al., 2013), suggesting that the likely degree of overlap would be high. Additional research is needed to examine whether there are differences in risk factors for those who have engaged only in suicidal or non-suicidal self-harm and those who have engaged in both behaviours.

The relationship between suicidal intent and self-harm frequency is likely to be complex. It is possible that those who have self-harmed on multiple occasions are more likely to have self-harmed with suicidal intent, and if so, this could provide an alternative explanation for some of the differences in associations found. However, a study by Hawton et al. (2010) found that adolescents who had cut themselves were more likely to self-harm repeatedly and were less likely to report suicidal intent than those who had self-poisoned. Information on lifetime frequency of self-harm was not available in this study, but data on past year frequency suggest that repetition of self-harm was more common amongst those who had harmed with suicidal intent. For most variables, our conclusions were generally unchanged when the sample was restricted to those who had harmed only once in the previous year (*n*=303, results available on request).

In addition, we did not investigate the possibility of confounding; however, it was not our aim to identify independent predictors of self-harm, and to examine this adequately would require a separate theory-driven analytical model for each exposure. This was beyond the scope of the current paper, but is an important area for future research. Finally, some measures were reported by mothers (e.g. sexual abuse) which may result in underestimates. Participants may also have failed to report self-harm, although this is less of an issue with self-report questionnaires than with interview-based measures (Evans et al., 2005)

### 4.3 Relevance to wider literature

Similar to Wichstrøm, 2009 we identified both common and specific risk factors for self-harm with and without suicidal intent, however, in contrast with his study, we found depression to be more strongly associated with suicidal self-harm. This finding is consistent with most previous literature (Wong et al., 2007, Larsson and Sund, 2008, Grøholt et al., 2000, Nock and Kessler, 2006, Cloutier et al., 2010, Jacobson et al., 2008) whereas previous research regarding anxiety (Wong et al., 2007, Larsson and Sund, 2008, Nock and Kessler, 2006, Wilkinson et al., 2011, Cloutier et al., 2010, Jacobson et al., 2008) and substance use (Nock and Kessler, 2006, Grøholt et al., 2000, Wichstrøm, 2009, Wong et al., 2007, Jacobson et al., 2008) has failed to produce any clear conclusions. Methodological differences in the operationalisation of substance use may go some way to explain the discrepant findings, for example, Wichstrøm, 2009 did not find substance use to differ according to suicidal intent, however, he used a combined measure that did not include smoking. Our results highlight the importance of distinguishing between different forms of substance use as a different pattern of association was found for smoking than for alcohol and cannabis use Many studies have found that smoking is associated with an increased risk of suicide/suicidal behaviour (Hughes, 2008), although the causal nature of this association is uncertain. The strong association of smoking with depression (Flensborg-Madsen et al., 2011, Lien et al., 2009) may underlie its stronger association with self-harm than that seen with alcohol and cannabis use in our study.

The concept of impulsivity is also heterogeneous and includes characteristics such as ‘disinhibition’, ‘sensation-seeking’ and ‘risk taking’ (Christiansen et al., 2012, Glenn and Klonsky, 2010). Higher levels of impulsivity have been found amongst those who self-harm with suicidal intent than without (Cloutier et al., 2010, Nock and Kessler, 2006) however, we found little evidence of an association in this study, possibly due to limitations of the measure that we used which was based on computer reaction times to visual stimuli (Glenn and Klonsky, 2010). In contrast, measures of sensation-seeking were found to be associated with self-harm, although the pattern was different for the two subscales of intensity-seeking and novelty-seeking. This suggests that these different aspects of impulsivity are related but distinct concepts.

The few (largely cross-sectional) studies that have investigated exposure to self-harm in others have similarly found stronger associations with suicidal than non-suicidal self-harm (Hargus et al., 2009, Larsson and Sund, 2008, Wong et al., 2007). For example, in their UK school study, Hargus et al. (2009) found that family suicide/self-harm distinguished between self-harm with and without suicidal intent for females and between acts of suicidal self-harm and thoughts for both genders. As found previously (Geulayov et al., 2014), the specific association between parent-reported suicide attempt and suicidal self-harm found in this study suggests there may be some specificity in transmission across generations. Associations here could be genetically mediated or could also indicate modelling effects; however, as this measure is maternally rated it is not clear whether the children were aware of their parents’ behaviour.

As previously shown (Page et al., 2013), lower socioeconomic position was more strongly associated with suicidal self-harm with little evidence of an association with non-suicidal self-harm. Some population studies have reported higher levels of suicide and suicidal behaviour amongst those with lower socioeconomic position (Qin et al., 2003, Fergusson et al., 2000); however, a review by Evans et al. (2004) found little evidence of an association in adolescents.

### 4.4 Summary

Self-harm with and without suicidal intent may be distinguished in terms of their relationship with a number of recognised risk factors, suggesting that these are overlapping behaviours, but with some distinct characteristics. While recognising limitations inherent in assessing suicidal intention, this distinction may be important for both research and clinical practice. Further research is needed to investigate whether these behaviours have different clinical outcomes, which may help to inform risk assessment, treatment and prevention efforts.

## Role of funding source

This research was funded by the 10.13039/501100000265Medical Research Council to study the causes and consequences of self-harm with and without suicidal intent in adolescence (principal investigator, David Gunnell; grant reference MR/J012661/1).

The UK Medical Research Council and the Wellcome Trust (Grant ref: 092731) and the University of Bristol provide core support for ALSPAC. This publication is the work of the authors who serve as guarantors for the contents of this paper. The study sponsor had no further role in the study design and collection, analysis and interpretation of data or in the writing of the article and the decision to submit it for publication.

DG, KH and GL are National Institute for Health Research (NIHR) Senior Investigators

## Conflict of interest

None of the authors report any conflict of interest

## Footnotes

Appendix A Supplementary data associated with this article can be found in the online version at doi:10.1016/j.jad.2014.07.009.
