# Supplementary material for: Differences in risk factors for self-harm with and without suicidal intent: Findings from the ALSPAC cohort
Source: J Affect Disord. 2014 Oct 15;168:407–14. doi: 10.1016/j.jad.2014.07.009 (PMC4160300; doi:10.1016/j.jad.2014.07.009)
Supplement: Supplementary file 1 — Supplementary Data [file mmc1.pdf]

Supplementary Table 1: Associations between exposures and self-harm with and without suicidal intent. A comparison of complete case and multiple imputation analysis

Exposure	Self-harm without suicidal intent versus no self-harm		Self-harm with suicidal intent versus no self-harm		Self-harm with suicidal intent versus self-harm without suicidal intent	
	Complete case	Multiple imputation	Complete case	Multiple imputation	Complete case	Multiple imputation
	OR [95% CI]	OR [95% CI]	OR [95% CI]	OR [95% CI]	OR [95% CI]	OR [95% CI]
Female gender [a]	3.28 [2.65, 4.05]	3.28 [2.65, 4.05]	3.68 [2.77, 4.90]	3.68 [2.77, 4.90]	1.12 [0.80, 1.58]	1.12 [0.80, 1.58]
Socioeconomic position						
Equivalised income (per quintile, 0 [high] -4 [low])	1.02 [0.95, 1.09]	1.01 [0.95, 1.08]	1.19 [1.09, 1.30]	1.21 [1.11, 1.31]	1.17 [1.06, 1.30]	1.19 [1.07, 1.32]
Parent social class (other vs. professional/managerial)	0.95 [0.78, 1.16]	0.95 [0.78, 1.15]	1.43 [1.12, 1.82]	1.44 [1.13, 1.83]	1.50 [1.12, 2.01]	1.52 [1.14, 2.03]
Mothers education (degree = reference)						
A level	0.69 [0.53, 0.90]	0.69 [0.53, 0.90]	1.50 [1.03, 2.20]	1.48 [1.01, 2.17]	2.17 [1.40, 3.37]	2.14 [1.38, 3.32]
O-level	0.93 [0.73, 1.18]	0.92 [0.73, 1.17]	1.61 [1.11, 2.33]	1.59 [1.10, 2.30]	1.74 [1.14, 2.63]	1.72 [1.14, 2.62]
<O-level	0.64 [0.48, 0.86]	0.65 [0.48, 0.86]	1.64 [1.10, 2.44]	1.66 [1.12, 2.47]	2.54 [1.59, 4.06]	2.57 [1.61, 4.09]
Total IQ (10 point increments)	1.13 [1.06, 1.21]	1.14 [1.07, 1.21]	0.93 [0.86, 1.01]	0.92 [0.85, 0.99]	0.83 [0.75, 0.91]	0.81 [0.74, 0.89]
Childhood sexual abuse	1.98 [0.71, 5.54]	2.32 [0.89, 6.01]	2.75 [0.90, 8.42]	3.54 [1.24, 10.1]	1.38 [0.37, 5.19]	1.53 [0.45, 5.17]
Cruelty to children in household	1.39 [0.84, 2.32]	1.38 [0.85, 2.24]	3.49 [2.16, 5.65]	3.26 [2.09, 5.09]	2.51 [1.34, 4.70]	2.36 [1.32, 4.24]
Being Bullied	1.51 [1.21, 1.88]	1.49 [1.19, 1.85]	2.33 [1.77, 3.07]	2.41 [1.85, 3.14]	1.55 [1.12, 2.14]	1.62 [1.18, 2.22]

Impulsivity (stop-signal task)	1.00 [0.96, 1.04]	1.00 [0.96, 1.05]	0.67 [0.92, 1.01]	0.97 [0.92, 1.01]	0.96 [0.91, 1.02]	0.96 [0.91, 1.02]
Sensation-seeking (5 point increments)						
Arnett intensity subscale	1.47 [1.32, 1.64]	1.48 [1.32, 1.65]	1.54 [1.34, 1.77]	1.54 [1.33, 1.77]	1.04 [0.88, 1.23]	1.04 [0.88, 1.23]
Arnett novelty subscale	1.42 [1.28, 1.59]	1.43 [1.28, 1.59]	1.12 [0.97, 1.28]	1.13 [0.99, 1.29]	0.78 [0.67, 0.92]	0.79 [0.67, 0.93]
Body dissatisfaction	1.95 [1.60, 2.38]	1.92 [1.57, 2.35]	2.85 [2.21, 3.69]	2.84 [2.20, 3.66]	1.46 [1.08, 1.98]	1.48 [1.09, 1.99]
Mental Health						
Depressive symptoms (SMFQ 11+)	2.67 [2.04, 3.51]	2.63 [2.03, 3.40]	5.08 [3.72, 6.93]	4.97 [3.70, 6.69]	1.90 [1.32, 2.72]	1.89 [1.34, 2.66]
DAWAB depression	2.73 [1.31, 5.71]	2.14 [1.06, 4.30]	9.05 [4.74, 17.3]	7.47 [4.10, 13.6]	3.31 [1.53, 7.16]	3.50 [1.64, 7.43]
DAWBAAB anxiety	2.42 [1.20, 4.86]	2.06 [1.08, 3.92]	7.37 [3.95, 13.7]	7.20 [4.07, 12.7]	3.05 [1.44, 6.46]	3.50 [1.72, 7.13]
Substance use						
Alcohol (heavy drinking)	1.98 [1.56, 2.52]	1.92 [1.52, 2.43]	1.67 [1.20, 2.32]	1.71 [1.25, 2.34]	0.84 [0.58, 1.23]	0.89 [0.62, 1.28]
Cannabis (occasional)	3.58 [2.63, 4.86]	3.21 [2.38, 4.33]	2.82 [1.85, 4.28]	2.38 [1.62, 3.51]	0.79 [0.50, 1.24]	0.74 [0.48, 1.14]
Smoking (weekly)	2.09 [1.48, 2.94]	1.59 [1.17, 2.16]	4.87 [3.41, 6.96]	3.51 [2.53, 4.88]	2.34 [1.52, 3.59]	2.21 [1.49, 3.29]
Self-harm in friends and family						
Parent suicide attempt	0.82 [0.32, 2.09]	0.90 [0.36, 2.24]	4.20 [2.23, 7.92]	4.24 [2.31, 7.81]	5.15 [1.83, 14.4]	4.74 [1.73, 13.0]
Mother self-harm (child-rated)	3.50 [1.82, 6.72]	3.41 [1.78, 6.55]	12.2 [6.95, 21.3]	11.9 [6.82, 20.9]	3.48 [1.83, 6.62]	3.50 [1.84, 6.66]
Father self-harm (child-rated)	1.51 [0.56, 4.09]	1.50 [0.55, 4.07]	4.28 [1.84, 9.98]	4.26 [1.83, 9.93]	2.84 [0.92, 8.76]	2.84 [0.92, 8.75]
Friend self-harm (child-rated)	5.87 [4.76, 7.24]	5.86 [4.75, 7.22]	7.69 [5.75, 10.3]	7.70 [5.76, 10.3]	1.31 [0.93, 1.84]	1.31 [0.93, 1.85]

[a] *Gender contains no missing values*
All analyses adjusted for participant gender
SMFQ: short mood and feelings questionnaire

Supplementary Table 2: Gender-exposure interactions and gender stratified results

Gender*Exposure interaction [a]	Interaction p-value	Self-harm without suicidal intent versus no self-harm		Self-harm with suicidal intent versus no self-harm	
		Male	Female	Male	Female
Socioeconomic position					
Equivalised income (per quintile, 0 [high] -4 [low])	0.562	0.97 [0.84, 1.11]	1.04 [0.96, 1.12]	1.12 [0.93, 1.35]	1.21 [1.10, 1.34]
Parent social class (other vs. professional/managerial)	0.111	0.85 [0.56, 1.29]	0.99 [0.80, 1.24]	0.85 [0.48, 1.52]	1.62 [1.24, 2.13]
Mothers education (degree = reference)	0.256	Reference	Reference	Reference	Reference
A level		0.48 [0.28, 0.80]	0.79 [0.58, 1.07]	1.00 [0.47, 2.12]	1.74 [1.12, 2.72]
O-level		0.74 [0.47, 1.17]	1.01 [0.76, 1.34]	1.36 [0.67, 2.73]	1.74 [1.13, 2.68]
<O-level		0.41 [0.21. 0.81	0.74 [0.53, 1.04]	0.74 [0.29, 1.91]	2.00 [1.27, 3.16]
Total IQ (10 point increments)	0.326	1.20 [1.05, 1.36]	1.11 [1.03, 1.19]	1.01 [0.86, 1.19]	0.91 [0.82, 1.00]
Childhood sexual abuse	-	-	2.67 [0.89, 8.01]	-	3.63 [1.11, 11.9]
Cruelty to children in household	0.862	1.29 [0.50, 3.31]	1.42 [0.77, 2.62]	4.21 [1.79, 9.86]	3.24 [1.81, 5.79]
Being Bullied	0.138	1.28 [0.80, 2.04]	1.60 [1.25, 2.07]	1.42 [0.77, 2.61]	2.69 [1.97, 3.67]
Impulsivity (stop-signal task)	0.600	1.01 [0.93, 1.10]	1.00 [0.95, 1.05]	0.93 [0.86, 1.01]	0.98 [0.93, 1.04]
Sensation-seeking (5 point increments)					
Arnett intensity subscale	0.700	1.45 [1.16, 1.82]	1.49 [1.31, 1.68]	1.37 [1.00, 1.86]	1.58 [1.35, 1.85]

Arnett novelty subscale	0.122	1.71 [1.35, 2.18]	1.35 [1.20, 1.53]	1.32 [0.96, 1.82]	1.07 [0.92, 1.24]
Body dissatisfaction	0.983	1.98 [1.29, 3.06]	1.95 [1.56, 2.43]	2.98 [1.72, 5.15]	2.82 [2.11, 3.77]
Mental Health					
Depressive symptoms (SMFQ 11+)	0.160	3.90 [2.16, 7.04]	2.48 [1.83, 3.36]	3.30 [1.50, 7.27]	5.48 [3.88, 7.74]
DAWAB depression	-	-	3.01 [1.40, 6.51]	15.5 [3.74, 63.9]	8.28 [4.06, 16.9]
DAWBAAB anxiety	-	2.21 [0.27, 18.2]	2.55 [1.21, 5.38]	-	8.60 [4.45, 16.6]
Substance use					
Alcohol (heavy drinking)	0.139	1.84 [1.10, 3.05]	2.05 [1.56, 2.70]	0.81 [0.34, 1.94]	1.97 [1.37, 2.83]
Cannabis (occasional)	0.124	4.91 [2.93, 8.21]	3.13 [2.14, 4.58]	1.62 [0.63, 4.22]	3.20 [1.99, 5.14]
Smoking (weekly)	0.004	2.95 [1.49, 5.83]	1.97 [1.33, 2.92]	0.93 [0.22, 3.95]	6.09 [4.13, 9.00]
Self-harm in friends and family					
Parent suicide attempt	0.748	0.76 [0.10, 5.70]	0.81 [0.28, 2.35]	6.12 [2.02, 18.5]	3.62 [1.70, 7.72]
Mother self-harm (child-rated)	-	-	4.49 [2.20, 9.15]	9.31 [2.50, 34.7]	13.8 [7.25, 26.2]
Father self-harm (child-rated)	-	2.20 [0.27, 18.0]	1.44 [0.47, 4.43]	-	5.07 [2.08, 12.3]
Friend self-harm (child-rated)	0.004	9.07 [6.07, 13.5]	4.93 [3.88, 6.28]	13.2 [7.39, 23.7]	6.27 [4.52, 8.69]

SMFQ: short mood and feelings questionnaire

[a] *Some models cannot be run for males for as there is one cell with a '0' value*
